# Automated particle recognition for engine soot nanoparticles

**DOI:** 10.1111/jmi.13140

**Published:** 2022-09-16

**Authors:** E. Haffner‐Staton, L. Avanzini, A. La Rocca, S. A. Pfau, A. Cairns

**Affiliations:** ^1^ Department of Mechanical, Materials and Manufacturing Engineering University of Nottingham University Park Nottinghamshire UK

**Keywords:** automotive, nanoparticles, neural networks, soot, TEM, vision learning

## Abstract

A pre‐trained convolution neural network based on residual error functions (ResNet) was applied to the classification of soot and non‐soot carbon nanoparticles in TEM images. Two depths of ResNet, one 18 layers deep and the other 50 layers deep, were trained using training‐validation sets of increasing size (containing 100, 400 and 1400 images) and were assessed using an independent test set of 200 images. Network training was optimised in terms of mini‐batch size, learning rate and training length. In all tests, ResNet18 and ResNet50 had statistically similar performances, though ResNet18 required only 25–35% of the training time of ResNet50. Training using the 100‐, 400‐ and 1400‐image training‐validation sets led to classification accuracies of 84%, 88% and 95%, respectively. ResNet18 and ResNet50 were also compared for their ability to categorise soot and non‐soot nanoparticles via a fivefold cross‐validation experiment using the entire set of 800 images of soot and 800 images of non‐soot. Cross‐validation was repeated 3 times with different training durations. For all cross‐validation experiments, classification accuracy exceeded 91%, with no statistical differences between any of the network trainings. The most efficient network was ResNet18 trained for 5 epochs, which reached 91.2% classification after only 84 s of training on 1600 images. Use of ResNet for classification of 1000 images, the amount suggested for reliable characterisation of soot sample, requires <4 s, compared with >30 min for a skilled operator classifying images manually. Use of convolution neural networks for classification of soot and non‐soot nanoparticles in TEM images is highly promising, particularly when manually classified data sets have already been established.

## 1 INTRODUCTION

Incomplete combustion of hydrocarbon fuel in internal combustion engines (ICEs) lead major fraction of exhaust particle matter (PM),^1^ accounting for 40% to >90% of the total PM depending on operating conditions.^2^ Due to severe negative effects on health and environment, PM has been limited by the European Union emission standards in terms of both particle mass and particle number since 2011.^3^ Despite trends towards electrified powertrains and decarbonised fuels, continued improvement and optimisation of ICEs still appear the best short‐term solution in terms of affordability, energy security and impact of greenhouse gas emissions.^4^ Decarbonisation of haulage/shipping faces difficulties in terms of performance and infrastructure requirements, such that the UK's ‘core’ NetZero strategy suggests only 13% of surface transport fleet will be decarbonised by 2050.^5^ As such, it is likely that engine PM emissions will remain an important target for regulations for the next few decades at least.

Soot emissions from combustion are a major concern for both human health and climate change. Atmospheric carbon particles are considered second only to CO_2_ in terms of contribution to the greenhouse effect and have been shown to impact respiratory and cardiovascular health,^6^ while also being linked to cancer and impaired neurovascular function.^7^ The morphology of soot plays a critical role in determining their effects on human and environmental health. It has been observed that the fractal morphology of soot^8^ and its hygroscopic properties^9^ influence the depth of inhalation into the lungs, and the surface area of soot nanoparticles has been correlated to their toxicity.^10^ The fractal morphology of soot has been seen to influence refractive properties in the atmosphere,^11^ influencing light adsorption and contribution to the greenhouse effect. As such, characterisation of soot morphology has been an important topic of research over the last few decades, with a view to more deeply understanding these soot‐related phenomena.^12^

Due to the nanoscopic size of soot (50–500 nm),^13^, ^14^ transmission electron microscopy is required for imaging. Transmission electron microscopy (TEM) has been widely used to characterise properties such as maximum length, fractal dimension, radius of gyration and primary particle diameter.^13^, ^15^, ^16^, ^17^, ^18^ However, TEM produces 2D projections while soot nanoparticles are known to be complex and highly irregular 3D structures, leading to significant errors.^17^, ^19^, ^20^, ^21^, ^22^ Electron tomography (ET) has been used to create 3D reconstructions of soot nanoparticles from a series of 2D TEM images acquired over a wide range of angles (e.g. ±60°).^17^, ^20^, ^21^, ^22^, ^23^, ^24^, ^25^ ET can produce highly accurate 3D models of soot nanoparticles but is significantly slower due to each particle requiring multiple images, and suitable particles being more difficult to locate on a TEM grid. Work by our research group has aimed to optimise the ET process for soot by implementing automated image acquisition and processing to identify suitable particles over large areas (∼40 μm^2^) of the TEM grid.^17^, ^19^, ^23^ This procedure also permits rapid measurement of particles in 2D, allowing greatly increased sample sizes and thus increased reliability of TEM‐derived measurements.^26^ However, due to low contrast of soot against the TEM grid substrate, and high concentrations of non‐soot particles in samples, a manual review process was required to separate real soot structures from false positives.^17^, ^23^

Deep learning is a statistical technique, which uses data to classify patterns using neural networks.^27^ It can be applied to automate the manual particle detection procedure, classifying nanoparticles in a few seconds, reducing operational time and contributing significantly to the increased throughput of ET for soot. Artificial neural networks (ANNs) are deep learning architectures made of neurons, which receive inputs from other neurons or external sources (e.g. pictures, words), weight them using kernels and compare to a threshold value in order to categorise them. Analysing images is the key to study soot; however, to process an image using a simple artificial neural network would require it to be converted into a 1D vector. This approach does not take into account important features such as pixel arrangements in corners, which helps to differentiate an image from another.^28^ As a result, convolutional neural networks (CNNs) are used to analyse and process images by detecting key pattern parameters and are used in software, which classifies images.^29^ The machine learning tool detects and segments patterns on the images and learns from them, making the algorithm increasingly accurate as more images are used for training.^30^

Examples of CNNs in research include segmentation of magnetic resonance images of the brain^31^ and identification of ferrography wear particles through image recognition to determine failure modes of metals depending on the shape of wear particles.^32^ Additionally, Raghu et al. used pre‐trained CNN architectures to classify seizure types.^33^ The technique used is called ‘transfer learning’ and it consists of training a pre‐trained architecture,^34^ which was originally trained to recognise images of 1000 categories, to recognise images of the topic of interest. ResNet, AlexNet, GoogleNet, VGG and SqueezeNet were the pre‐trained CNN architectures investigated.^33^ It has been observed that deeper networks can be subject to degradation problems when they converge: as size increases, accuracy gets saturated and degrades rapidly.^35^ Hence, an alternative approach to address the problem is formulating an architecture based on residual functions. While stacked convolutional layers learn features, residual learning uses residual errors to study patterns. The pre‐trained CNN architecture ‘ResNet’ does this by subtracting features which are learned by the input layer, allowing the software to be more easily trainable and to be subject to much less degradation when layers are increased.^36^ ResNet is pre‐trained to learn 1000 categories of images to be able to classify them. The architecture can be adapted by replacing the final layer before the fully connected layers (called ‘fc1000’), with a new layer which only contains categories of interest. Xiang et al. compared ResNet18, ResNet50 and ResNet101 to identify the ideal ResNet architecture to classify two categories of images, with ResNet18 found to be the more accurate despite being the shallower network.^37^ Nibali et al. used pre‐trained ResNet18 to classify images of Pulmonary nodule,^38^ in a study with similar scope to this work.

In this study two depths of ResNet, one 18 layers deep (ResNet18) and one 50 layers deep (ResNet50), were applied to the problem of classifying soot and non‐soot carbon nanoparticles TEM images from a sample of soot‐in‐oil taken from a GDI passenger vehicle. The two networks were first assessed using training‐validation sets of increasing size: containing 100, 400 and 1400 images. Though generally an increase in network accuracy with increasing training‐validation set size is expected,^39^ the rate of this increase is of interest. Due to strong differences in the composition of different soot samples (e.g. gasoline soot‐in‐oil vs. diesel exhaust soot), it is possible that separate network trainings may be required for different types of soot on a per‐sample basis. As such, small training‐validation sets may be the only available training resource. Alternatively, similar soot samples (such as those from repeated experiments of the same soot‐in‐oil) may benefit from training networks on one another's data, so larger training‐validation sets are also of interest for a ‘best‐case’ scenario. ResNet18 and ResNet50 were also compared for soot/non‐soot classification through a fivefold cross‐validation experiment using 1600 images (with a 1:1 ratio of soot and non‐soot images). Automated classification of soot and non‐soot carbon nanoparticles would not only greatly increase throughput and reliability of soot morphology characterisations, where upwards of 1000 nanoparticles need to be measured,^26^ but also permits future studies on the non‐soot fraction of nanoparticles in soot‐in‐oil samples, which at present are not well understood. Modifying the architecture of the pre‐trained software and training it to recognise specific classes of interest has the potential to be applied widely in the field of nanoparticles research using TEM.

### 1.1 Methodology

**FIGURE 1:**
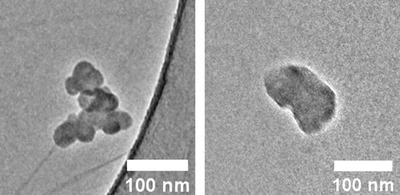
(Left) Typical soot‐in‐oil nanoparticle imaged at ×10,000 magnification. Note the clear presence of spherical ‘primary particle’ sub‐units around 30 nm in diameter, and varied grey‐levels within the structure due to overlapping of primary particles. (Right) Typical amorphous carbon ‘non‐soot’ nanoparticle from the same soot‐in‐oil sample. Note lack of primary particle sub‐units, less complex and more rounded structure, and more consistent contrast/grey‐levels due to more uniform structure

Nanoparticles were extracted from a sample of soot‐laden oil taken from a passenger GDI vehicle, via a solvent dilution process using heptane and diethyl ether (further details can be found in a previous study).^40^ Nanoparticles were prepared for TEM by deposition on 300‐mesh copper TEM grids coated with graphene oxide on lacey carbon. Previous study of this sample revealed high concentrations of carbonaceous nanoparticles of similar size and density/contrast soot, but which lack the characteristic fractal‐like morphology, primary particle sub‐units and graphitic nanostructure of soot. An example of a soot nanoparticle and non‐soot amorphous carbon nanoparticle is shown in Figure 1. These non‐soot carbon nanoparticles have been referred to as amorphous carbon or sludge‐like nanoparticles in previous studies of soot‐in‐oil,^41^ but as yet are not well understood. The input data (i.e. TEM images) for the present work is the same used in the development of the semi‐automated procedure,^40^ from which a total of 800 images of soot and 800 images of non‐soot nanoparticles were collected.

**FIGURE 2:**
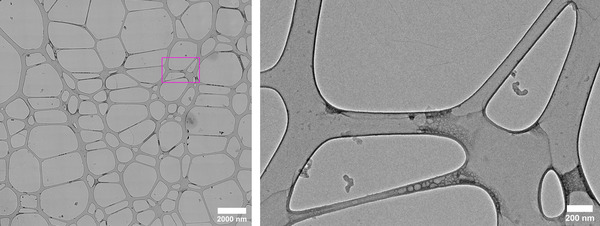
Example of a large‐scale stitched montage used for mapping (left) and zoome section showing soot nanoparticles at ×10,000 magnification (right)

The procedure for rapid soot identification from our previous work was based upon an automated TEM mapping and image processing. The open‐source TEM control software SerialEM was used to create large‐scale (approximately 20 μm^2^) maps of the surface of the TEM grid via ‘montaging’.^42^ Montaging works by acquiring a series of overlapping images in a grid‐like pattern over a pre‐defined area, and cross‐correlating these images to stitch them into a single large image (a ‘montage’, see Figure 2). Several easily identified features of the stitched montage are then aligned to their absolute position in the microscope coordinate system, and a ‘montage map’ is created. Features of interest in the montage map can then be selected and the microscope is able to automatically locate that feature in the live image feed for subsequent tilt‐series acquisition.

**FIGURE 3:**
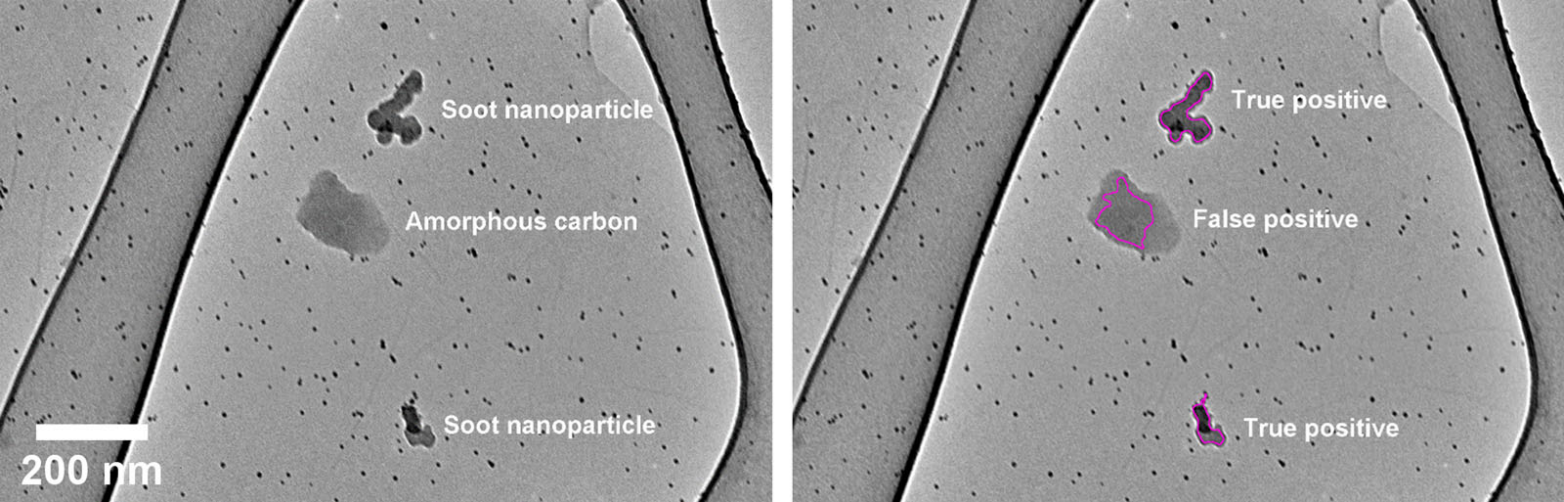
(Left) Sub‐section from a montage map showing two soot nanoparticles, one globular amorphous carbon structure, and unclustered gold nanoparticles in the background. (Right) Automated selections from the image processing algorithm, showing two true‐positive selections of the soot nanoparticles, and a false‐positive selection of the amorphous carbon

**FIGURE 4:**
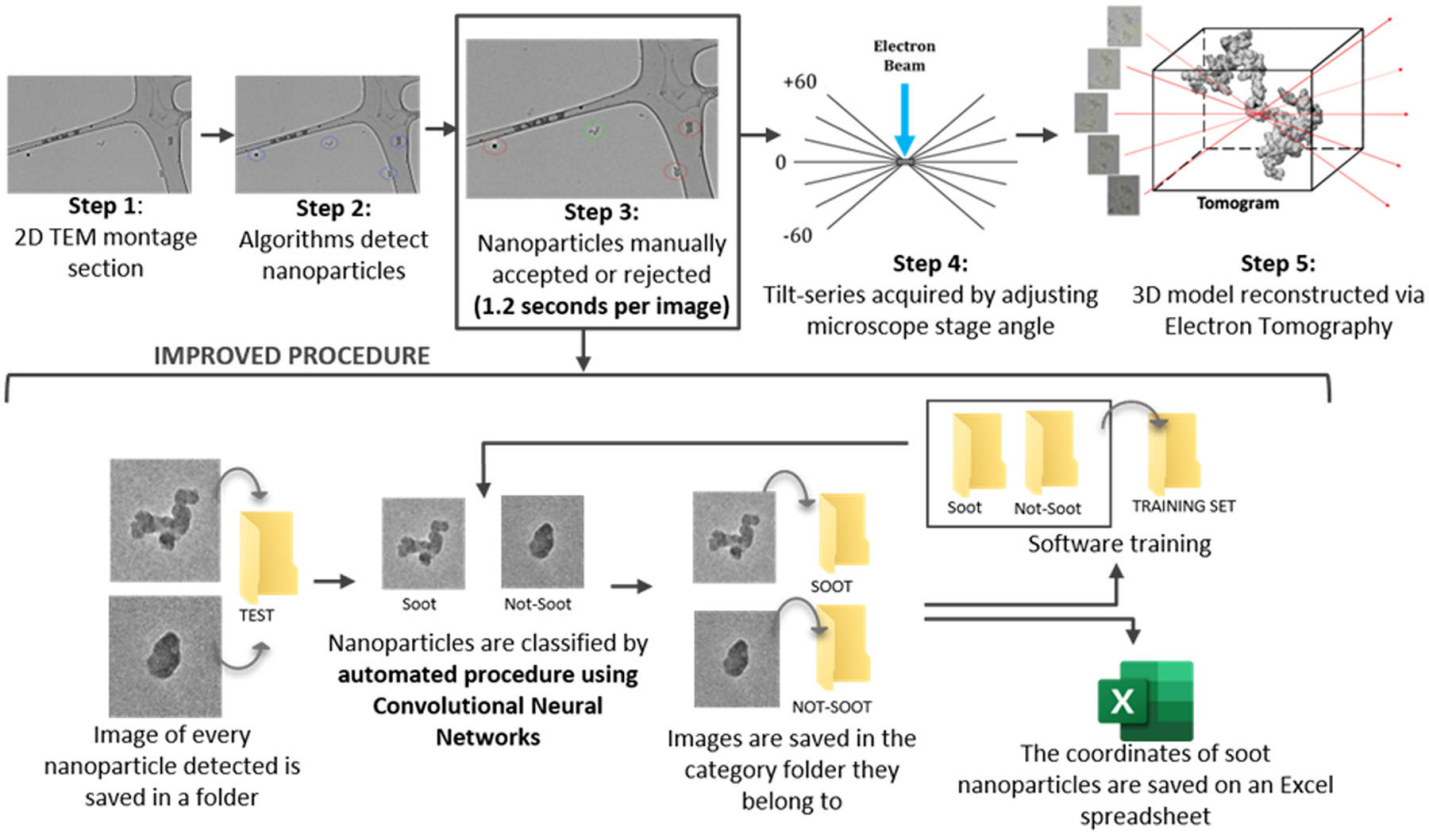
Current and improved procedure to rapidly identify and reconstruction soot nanoparticles in 3D. Details of electron tomography procedure in steps 4 and 5 can be found in prior publications^19^ ^,^ ^24^

To identify features of interest, the montage maps are rapidly processed using an automated image processing algorithm implemented in ImageJ. A series of filtering and thresholding processes, along with constraints on selection size and shape, are applied to automatically identify potential nanoparticles. In practice, however, imaging of soot extracted from engine lubricant oil was prone to false‐positives due to the presence of the non‐soot amorphous carbon nanoparticles (see Figure 3). Hence, a manual review process by the human operator was required to separate selections of soot nanoparticles and non‐soot nanoparticles. ResNet is used in this study as a potential replacement for the manual review process, and its integration into the automated soot characterisation procedure is summarised in Figure 4.

ResNet training and testing computations were carried out on MATLAB using an NVIDIA GeForce GTX 1650 GPU. The architecture of ResNet was modified to replace the fully connected layer with a layer containing pre‐classified images of soot and non‐soot nanoparticles (i.e. the training‐validation set). The image input size to ResNet is 224 × 224 pixels,^33^ as a result, both the images used for training and the images used for testing (analysis) must have a squared shape to preserve the aspect ratio once the image sizes are converted. Trained networks were testing by being used to classify an independent test set containing 100 images of soot and 100 images of non‐soot nanoparticles. The primary metrics used to determine the quality of test set classification were accuracy and F1 score:

(1) $$\mathrm{Accuracy}=\frac{\mathrm{TP}+\mathrm{TN}}{\mathrm{TP}+\mathrm{FN}+\mathrm{FP}+\mathrm{TN}},$$

(2) $$F1\mathrm{score}=\frac{2\;\times \;\mathrm{TP}}{2\;\times \;\mathrm{TP}+\mathrm{FN}+\mathrm{FP}},$$

where the true positive (TP) is the number of soot images correctly classified as soot. The true negative (TN) is the number of non‐soot images correctly classified as non‐soot. The false positive (FP) is the number of non‐soot images incorrectly classified as soot. The false negative (FN) is the number of soot images incorrectly classified as non‐soot. Table 1 shows how the values relate to each other.

**TABLE 1:** Confusion matrix

	Actual category	
Predicted category	Soot	Non‐soot
Soot	True positive	False positive
Non‐soot	False negative	True negative

Accuracy indicates the overall performance in detecting the true positive (TP) and the true negative (TN), i.e. correctly classifying soot and correctly classifying non‐soot. Accuracy is equally balanced between TP and TN classifications, so would not indicate if a network is much better at classifying non‐soot than soot, or vice versa. The F1 score is the harmonic mean of the precision (the proportion of ‘soot’ classifications that are actually soot nanoparticles) and the recall (the percentage of soot nanoparticles that were correctly classified as ‘soot’), and thus is weighted towards TP selections. In a traditional soot sample characterisation, a higher F1 score would be preferred as correct soot classifications are the primary goal. However, as morphological characterisation of non‐soot nanoparticles is also of interest to our research, accuracy is considered as the most important metric.

## 2 RESULTS AND DISCUSSION

First, the performances of ResNet18 and ResNet50 were measured as a function of 3 training‐validation set sizes. As described in the methodology, an independent test set of 200 images was separated from the rest of the data for classification assessments. Three ‘training‐validation’ sets were created from the remaining 1400 images, containing 100, 400 and 1400 images each (a 1:1 ratio of soot and non‐soot images in all cases). The training‐validation split was 70:30 in all cases, the validation frequency was fixed at once per training epoch and the stochastic gradient descent with momentum (SGDM) solver was used. Optimisation of the network training settings was carried out via varying the mini‐batch size and the (constant) learning rate. Three mini‐batch sizes (8, 32, 64 images) and three constant learning rates (0.01, 0.001, 0.0001) were tested, and at least 3 repeat network trainings were carried out for each combination (see Table 2). These trainings were carried out for an initial 100 training epochs, with the number of training iterations varying depending on the mini‐batch size and training‐validation set size. Following identification of the optimal training settings, the number of epochs/iterations for network training was also optimised as detailed in the following section.

**TABLE 2:** Mini‐batch sizes and learning rates used in optimisation of network trainings settings

	Learning rate		
Mini‐batch Size	0.01	0.001	0.0001
8	8, 0.01	8, 0.001	8, 0.0001
32	32, 0.01	32, 0.001	32, 0.0001
64	64, 0.01	64, 0.001	64, 0.0001

Following the tests on the effect of training‐validation set sizes, ResNet18 and ResNet50 were ultimately compared for quality on the classification of soot and non‐soot nanoparticles via a fivefold cross‐validation. The total set of 1600 images was divided into 5 equal ‘folds’ of 320 images, each containing 160 randomly selected images of soot and non‐soot. For a single cross‐validation experiment, ResNet18 and ResNet50 were trained with this data 5 times, with each fold being used as the validation data once and as training data the other 4 times. At the end of training, the final accuracy measurements were taken by using the network to classify the validation set. The accuracy was then averaged over the 5 trainings to give the final accuracy of the network. For both ResNet18 and ResNet50, training during cross‐validation was carried out using the optimal training settings found for the 1400‐image training‐validation set. The fivefold cross‐validation experiment was repeated 3 times, with the networks being trained for 5, 20 and 50 epochs, respectively.

### 2.1 Performance as a function of training‐validation set size

**FIGURE 5:**
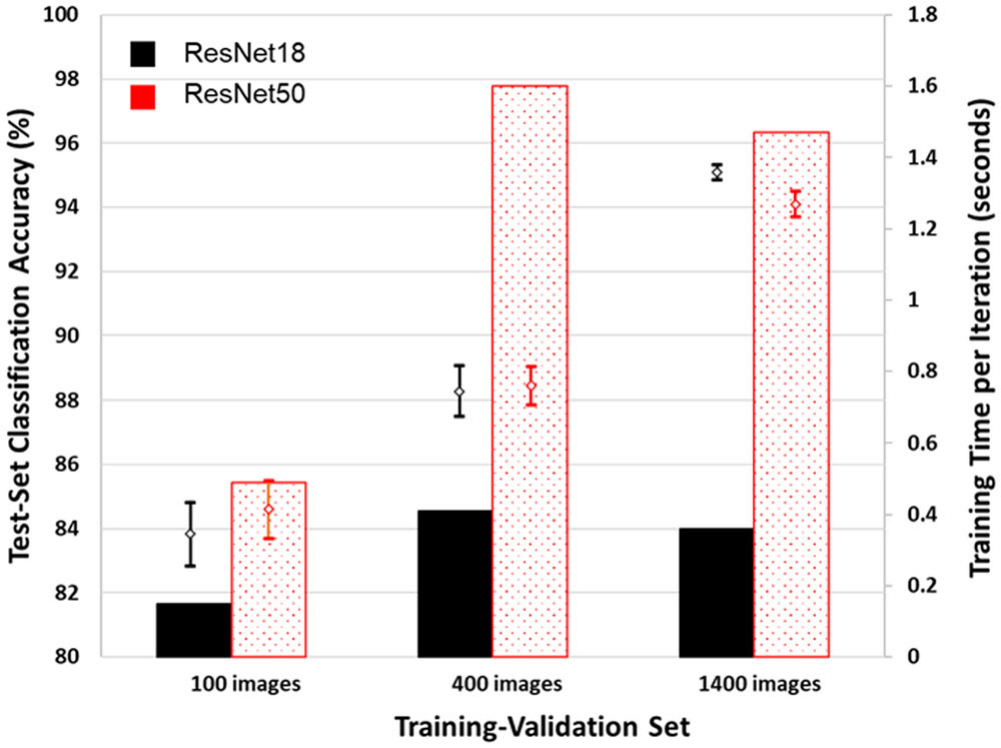
Optimum accuracies of ResNet18 and ResNet50 networks trained with training‐validation sets of increasing size, and training time required in terms of seconds per iteration. Error bars shown are 90% confidence intervals. Means and error bars are calculated from 9 repeated networks trainings in each

The optimal results from the network trainings using the 3 training‐validation sets are shown in Figure 5 and summarised in Table 3.

**TABLE 3:** Optimum results of ResNet18 and ResNet50 networks trained with training‐validation sets of increasing size

Network	Training‐validation images	Mini‐batch size	Learn rate	Training epochs	TP	TN	FP	FN	Accuracy (±90% CI)	*F*1 score (±90% CI)	Seconds per iteration
ResNet18	100	8	0.0001	200	77.0	90.7	9.3	23.0	83.8% (±1.0)	82.6 (±1.2)	0.15
ResNet50				200	78.0	91.2	8.8	22.0	84.6% (±0.9)	83.5 (±0.9)	0.49
ResNet18	400	32	0.0001	50	89.7	88.0	12.0	10.3	88.3% (±0.8)	88.2 (±0.8)	0.41
ResNet50				50	89.7	87.0	13.0	10.3	88.4% (±0.6)	88.5 (±0.6)	1.60
ResNet18	1400	32	0.001	50	92.7	97.5	2.5	7.3	95.1% (±0.2)	95.0 (±0.2)	0.36
ResNet50				50	94.2	94.0	6.0	5.8	94.1% (±0.4%)	94.1 (±0.4)	1.47

*Note*: Results shown are averages of 9 repeated trainings in all cases.

For the smallest training‐validation set, the optimal training settings were a mini‐batch size of 8 and a learn rate of 0.0001 for both ResNet18 and ResNet50. For all training‐validation set sizes, the combination of a 64‐image mini‐batch size and a learning rate of 0.0001 produced the lowest accuracies. Previous studies have observed inverse relationships between mini‐batch size and learning rate, with larger mini‐batch sizes observing improved training when used alongside larger learning rates and vice versa.^43^

**FIGURE 6:**
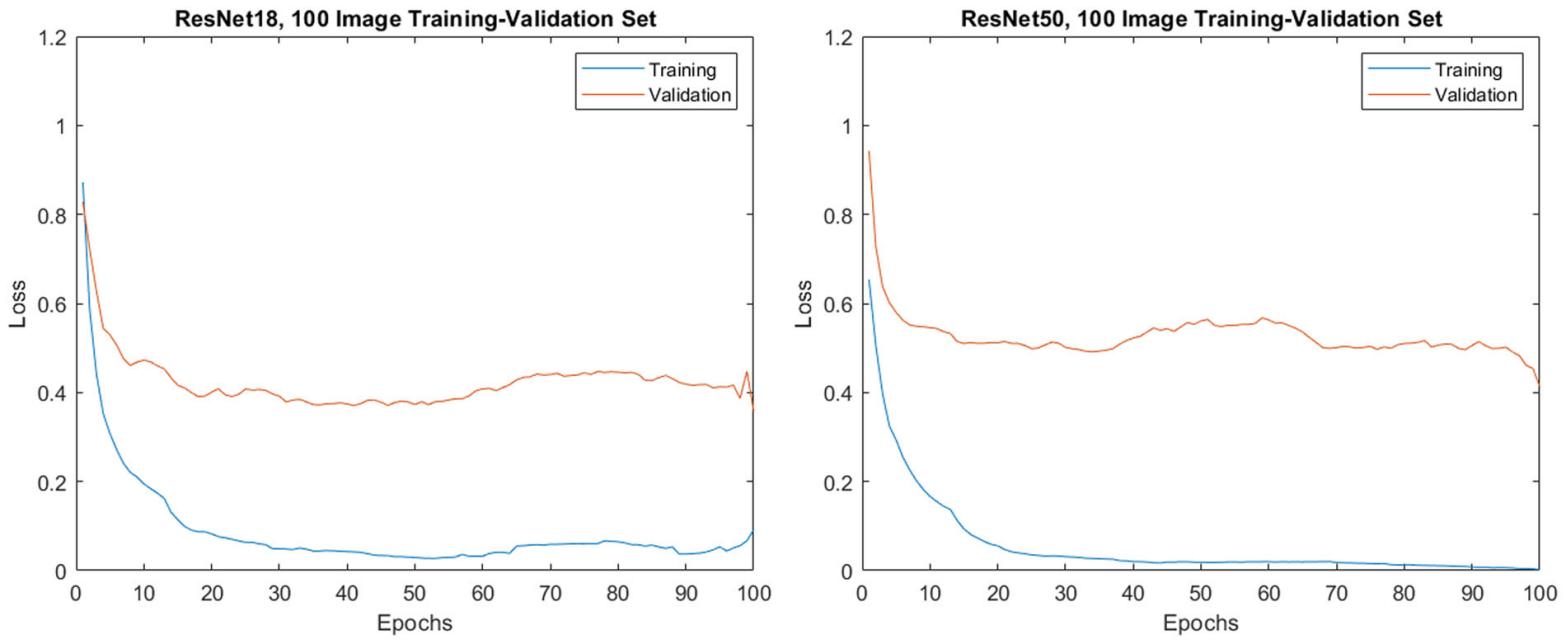
(Left) Training‐validation loss profile for ResNet18, trained for 100 epochs on the 100 image training‐validation set. (Right) Training‐validation loss profile for ResNet50, trained for 100 epochs on the 100 image training‐validation set. Note rapid initial decreases in both training and validation loss, followed by period of steady difference between training and validation loss, without noticeable increase in validation loss (i.e. overfitting)

Following the training settings optimisation, the training length was optimised by observing the training‐validation accuracy and loss values. The aim of training a network like ResNet is generalisation, i.e., producing a network that accurately classifies new data from outside of the training‐validation set. Typically, training will continue until a point at which ‘overfitting’ beings to occur, after which the network accuracy would start to decrease. Overfitting occurs when the network begins to ‘memorise’ features of the training images, at the expense of its ability to generalise. Overfitting is seen when improved classification of the training set occurs alongside decreased accuracy when classifying the validation set. One method to avoid overfitting and the associated generalisation errors is to perform early stopping,^44^ ceasing training at a point where the validation loss begins to increase above a certain threshold. Training‐validation loss curves for ResNet18 and ResNet50 trained on the 100‐image set are shown in Figure 6, where some interesting features can be observed.

**FIGURE 7:**
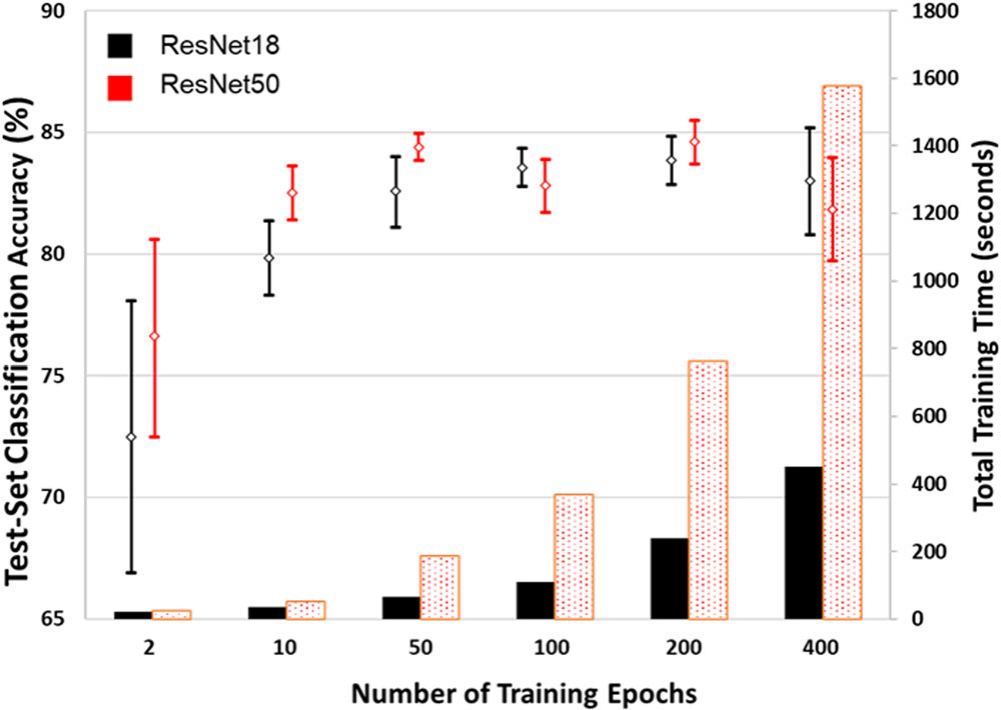
Effect of number of training epochs on the accuracy of networks trained on the 100‐image training‐validation set. Time required for training also shown

For the majority of network trainings in this study, the loss curves are similar to those shown in Figure 6. A decrease in both training and validation loss is strong during the first 5–10 epochs of training. After this point, there quickly becomes a deviation between training and validation loss, after which the separation between them remains relatively constant with increasing training duration. Though there are small fluctuations in the validation loss that occur there is generally no obvious onset of overfitting and as such no obvious point at which early stopping might be applied. To understand the effect of increasing training length on the accuracy of these networks, a series of tests were carried out at different training lengths. Training was repeated on the 100‐image set over a range of 2–400 epochs for both ResNet18 and ResNet50, results for which are shown in Figure 7 (where all network trainings were repeated at least 6 times).

For both ResNet18 and ResNet50 the highest average accuracy was observed after training for 200 epochs, at 83.8% and 84.3%, respectively (these results are quoted in Table 3). However, overlapping of the 90% confidence intervals occurred from 50 epochs onwards for ResNet18, and from 10 epochs onwards for ResNet50, showing that there was little benefit of increased training duration within the region of steady training‐validation loss difference. For each network, the training time varies approximately linearly with the number of training epochs. ResNet50 is around 3–4 times slower than ResNet18 for trainings from 50 epochs upwards. Even with the limited hardware used in this study, training times are modest.

For the 400‐image training‐validation set, the optimum training settings shifted to a mini‐batch size of 32, and a learning rate of 0.0001. Similar tests were carried out to understand the effect of training length, though were limited to 100 epochs following the observations for the 100‐image set that increased training length above 50 epochs did not lead to significant improvements in accuracy. An optimum training length of 50 epochs was observed and resulted in a test‐set classification accuracy of 88.3% for ResNet18 (in 162 s) and 88.4% for ResNet50 (in 641 s).

**FIGURE 8:**
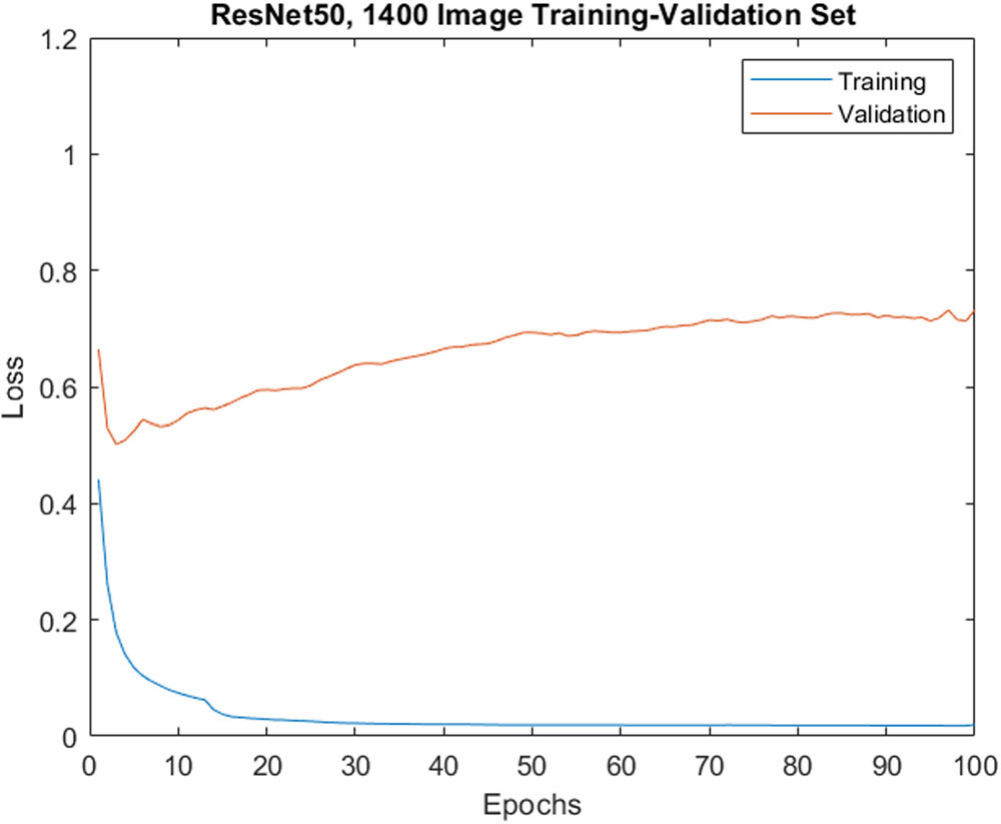
Training‐validation loss profile for ResNet50 trained on the 1400 image training‐validation set. Note the increase in validation loss occurring from around 2 epochs onwards (overfitting)

**FIGURE 9:**
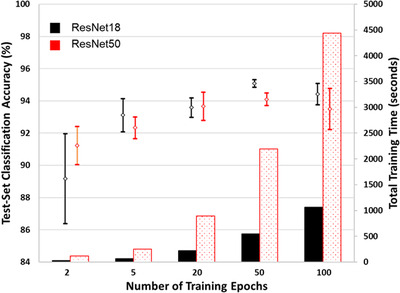
Effect of number of training epochs on the accuracy of networks trained on the 1400 image training‐validation set. Time required for training also shown

For the largest training‐validation set of 1400 images, the optimum training settings were a mini‐batch size of 32 and a learning rate of 0.001. For this set, the loss data for ResNet18 trainings again showed relatively steady validation loss with increased training duration. However, for ResNet50 the curves showed increasing validation losses occurring from around 2 epochs onwards (see Figure 8). Repeated network trainings were again carried out over a range of lengths, from 2 to 100 epochs. The effect of training length on the accuracy of networks for the 1400‐image set is shown in detail in Figure 9, where all network trainings were repeated at least 6 times.

For all trainings except the 2‐epoch training with ResNet18, test‐set classification accuracy was >90%. Both networks observe increasing accuracy up to 50 epochs of training, reaching 95.1% and 94.1% for ResNet18 and ResNet50, respectively, before decreasing slightly with a longer training duration of 100 epochs. There was no major difference in the effect of different training lengths on the accuracy of ResNet18 and ResNet50, despite the differences in the loss curves that were observed. As for the 100‐image set, training time varied approximately linearly with the number of training epochs, and ResNet50 was around 3–4 times slower than ResNet18.

**FIGURE 10:**
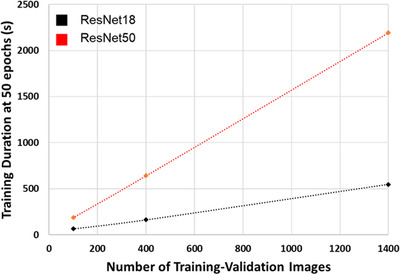
Network training time required for 50 epochs of training at the 3 training‐validation set sizes

As may have been expected, increasing the number of training‐validation images led to an increase in the accuracy of both ResNet18 and ResNet50 networks. Accuracy increased from 83–84% for a 100‐image set to 94–95% for a 1400‐image set. An interesting observation to note was that training with the 100‐image set led to networks that were better at classifying the non‐soot nanoparticles than the soot nanoparticles. The average number of correctly classified non‐soot nanoparticles in the test set (true negatives) was around 15% greater than the number of correctly classified soot nanoparticles (true positives). Networks trained on the larger training‐validation sets exhibited approximately equal numbers of true positives and true negatives, reflected in almost identical accuracies and *F*1 scores, suggesting similar ability to classify soot and non‐soot. As shown in Figure 10, when the number of training epochs was kept constant the time required for training varied approximately linearly with the number of training images. The time required per iteration of training did not vary linearly (see Table 3), as the number of iterations depends on the mini‐batch size which changed between the training‐validation sets.

Overall, performance of ResNet18 and ResNet50 was almost identical for each of the 3 training‐validation sets, though training of ResNet18 took only 25–35% of the time required for training ResNet50. Additionally, longer training durations were most beneficial for the 100‐image set, while training for longer than 5 epochs had only minor benefits for the 1400‐image set.

**FIGURE 11:**
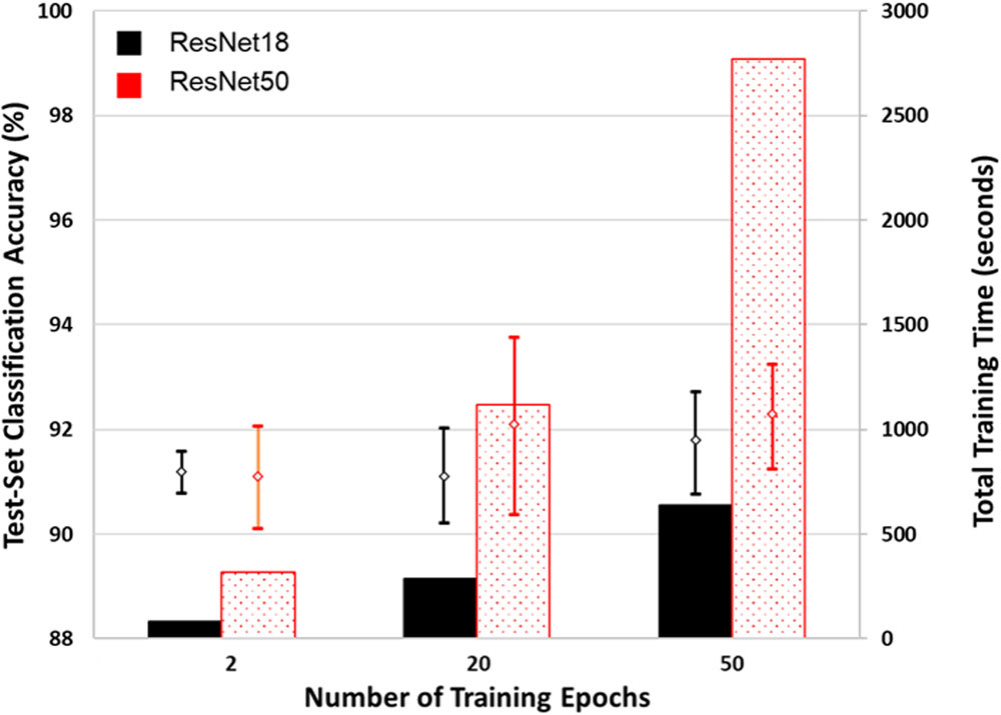
Results of fivefold cross‐validation experiment at 3 different training lengths

To ultimately compare the quality of ResNet18 and ResNet50 for the problem of soot sample classification, a fivefold cross‐validation experiment was carried out. The complete set of 1600 images was randomly divided into 5 ‘folds’ of 320 images, each containing 160 soot and non‐soot nanoparticles. Network training was carried out 5 times, with each fold acting as the validation set once and as a training set 4 times. Training was carried out using the mini‐batch and learn rate settings derived for the 1400‐image training‐validation set. The cross‐validation experiment was repeated with 3 different trainings lengths: 5, 20 and 50 epochs. Results are shown in Table 4 and summarised in Figure 11.

**TABLE 4:** Results of fivefold cross‐validation experiment at 3 different training lengths

	5 epochs		20 epochs		50 epochs	
Validation fold	ResNet18	ResNet50	ResNet18	ResNet50	ResNet18	ResNet50
1	91.7	93.4	91.9	94.4	91.6	93.4
2	90.0	90.6	90.6	91.6	90.9	91.6
3	91.6	90.2	91.6	90.6	92.8	90.9
4	91.4	91.9	91.9	93.4	92.8	93.1
5	91.3	89.4	89.7	90.3	90.6	92.2
Average accuracy %	91.2	91.1	91.1	92.1	91.8	92.3
Average duration (s)	84	318	285	1119	637	2771

All trainings in the cross‐validation experiment showed average accuracies above 91%, with no significant difference observed between ResNet18 and ResNet50. Additionally, the performance after just 5 epochs of training (150 iterations) produced networks of similar quality to 50 epochs of training. Based on these results, the optimum network for the classification of soot and non‐soot particles is ResNet18 trained for only 5 epochs, where training required only 84 s for 1600 images. The classification procedure required only 1.7 s for 320 images, which is of similar magnitude to manual classification of a single TEM image by a human operator, highlighting benefits to throughput when using CNNs for classification.

Overall, due to its faster training speed and similar levels of accuracy, ResNet18 would be considered the better network depth to use for the problem of classifying soot and non‐soot nanoparticles, especially in situations where available hardware is limited. Based on the accuracies measured, the use of the convolution neural network ResNet for the problem of soot/non‐soot classification is most applicable when a larger amount of training data is available (>400 images). This would equate to a scenario where a sample to be studied is similar to one imaged previously, for example, a repeat measurement or a sample derived from the same engine test, where set of training images would already exist. Applicability to novel samples is limited, as accuracies for a 100‐image training set (83–84%) would lead a significant number of incorrectly classified particles being measured and thus significant errors in the morphological characterisation of the sample. Based on the work of Kondo et al., it is suggested that 1000 individual nanoparticles are required to be measured to reliably characterise a sample of soot. Using a manual review process, a highly trained operator would require at least 30 min to categorise images whereas a pre‐trained network could complete this almost instantly (<4 s), with as little as 84 s required to train a network to >91% accuracy with a training‐validation set of >1000 images. Critically, automated identification significantly reduces the human labour cost and removes the need for a skilled operator, which is a key aspect of a true high‐throughput procedure.

## 3 CONCLUSIONS

This study focused on the development of an automated procedure for soot nanoparticle recognition, for incorporation into a previously developed semi‐automated procedure for soot analysis in 2D and 3D. By implementing ‘transfer learning’ on a pre‐trained convolutional neural network architecture, the 3D morphological reconstruction procedure of soot nanoparticles is made more efficient by reducing the time of operation. More specifically, CNNs are used to replace human supervision to classify the nanoparticles detected into ‘soot’ and ‘non‐soot’ categories, which can consequently be reconstructed in three dimensions. This work compared two depths of ResNet for the task of soot and non‐soot nanoparticle classification and included investigation of the effect of training‐validation set size on network accuracy.

‐ Accuracy of soot/non‐soot nanoparticle classification using ResNet increased with increasing number of training images. Training of ResNet with training‐validation set sizes of 100, 400 and 1400 images led to classifications accuracies of 83–84%, 88% and 94–95%, respectively.
‐ For all the training‐validation set sizes used in this study, ResNet18 and ResNet50 had almost identical classification accuracies, though ResNet18 required only 25–35% of the time required for training ResNet50.
‐ Training with a 100‐image training‐validation set produced networks significantly better at classifying non‐soot nanoparticles than soot nanoparticles. Training with larger sets of images produced networks with equivalent ability to categorise soot and non‐soot.
‐ Tests on the effect of training duration on network accuracy showed that optimal training length was short at around 50 epochs, with increased training durations leading to statistically insignificant differences in accuracy.
‐ Training time varied approximately linearly with number of training images and with number of training epochs. Training time per iteration depends on the network training settings.
‐ CNNs appear most suitable for soot/non‐soot classifications when >400 images that could be used for a training‐validation set already exist, for example, a repeat sample study or a sample derived from the same engine testing procedure.
‐ The fivefold cross‐validation experiment showed no significant differences in the accuracy of ResNet18 and ResNet50. No significant difference in accuracy was observed between networks trained for 5, 20 or 50 epochs, showing that optimal network training length was short.
‐ The optimal result of cross‐validation was ResNet18 trained for 5 epochs (on a 1600‐image training‐validation set). This training required only 84 s and reached 91.2% classification accuracy.
‐ Classification time using trained networks was negligible, requiring only 1.7 s for 320 images and <4 s for 1000 images. By comparison, classification by a human operator requires up to 2 s per single image, or >30 min to classify 1000 images (the amount suggested to be necessary for reliable morphology characterisations). Classification time is decreased by a factor of approximately 400–500 when using the automated procedure rather than the manual identification.
‐ Furthermore, since the training procedure does not need supervision, the software can run during low‐demand hours when trained with a large data set of thousands of images.
‐ The technology proves to be effective when combined with transmission electron microscopy, hence it can be adapted to further research tasks involving nanoparticles’ detection and TEM, opening a wide range of opportunities to apply pre‐trained convolutional neural networks by adopting the ‘transfer learning’ approach.
